# A Novel Lymphangiogenesis-Related Gene Signature can Predict Prognosis and Immunosuppressive Microenvironment in Patients with Clear Cell Renal Cell Carcinoma

**DOI:** 10.7150/ijms.81078

**Published:** 2023-04-24

**Authors:** Ke Chen, Mingchao Gao, Wen Dong, Hao Liu, Yi Lin, Yuxia Xie, Wenlong Zhong, Junyu Chen, Xiaodong Huang, Wang He, Tianxin Lin, Bo Wang, Jian Huang

**Affiliations:** 1 Department of Urology, Sun Yat-sen Memorial Hospital, Sun Yat-sen (Zhongshan) University, Guangzhou, China;; 2 Guangdong Provincial Key Laboratory of Malignant Tumor Epigenetics and Gene Regulation, Guangdong-Hong Kong Joint Laboratory for RNA Medicine, Medical Research Center, Sun Yat-Sen Memorial Hospital, Sun Yat-sen (Zhongshan) University, Guangzhou, China;; 3 Department of Urology, The Third Affiliated Hospital, Sun Yat-sen (Zhongshan) University, Guangzhou, China;; 4 Guangdong Provincial Clinical Research Center for Urological Diseases, Guangzhou, China.

**Keywords:** clear cell renal cell carcinoma, lymphangiogenesis-related genes, lymphangiogenesis, prognosis, tumor immune, T cell exhaustion.

## Abstract

**Background:** Lymphangiogenesis represents a key event in the progression and metastasis of patients with clear cell renal cell carcinoma (ccRCC). Nevertheless, the prognostic value of lymphangiogenesis-related genes (LRGs) in ccRCC patients remains unknown. **Method:** Differential analyses were performed to identify differentially expressed LRGs between normal and tumor tissues. A univariate Cox analysis was performed to identify differently expressed LRGs associated with overall survival (OS). LASSO and multivariate Cox analyses were performed to construct and optimize the LRG signature. To further explore the molecular characterization of the LRG signature, a functional enrichment analysis, immune signature, somatic mutations, and drug sensitivity were assessed. Immunohistochemistry (IHC) and immunofluorescence staining were performed to validate the relationship between lymphangiogenesis and immunity using our ccRCC samples. **Results:** Four candidate genes (IL4, CSF2, PROX1, and TEK) were eventually available to construct the LRG signature in the training set. Patients in the high-risk group had a shorter survival than those in the low-risk group. The LRG signature was an independent prognostic factor of OS. These results were confirmed in the validation group. The LRG signature was correlated with immunosuppressive cell infiltration, T cell exhaustion markers, somatic mutations, and drug sensitivity. The IHC and immunofluorescence staining results confirmed the correlation between lymphangiogenesis and CD163^+^ macrophages, exhausted CD8^+^PD-1^+^, and CD8^+^ LAG3^+^ T cells. **Conclusion:** A novel prognostic signature based on LRGs could provide insight into the prognostic evaluation and treatment of ccRCC patients.

## Introduction

Patients with clear cell renal cell carcinoma (ccRCC) are currently primarily treated with partial or total nephrectomy 1-3. Nevertheless, recurrence or metastasis still occurs in approximately 30% of patients following surgical procedures 4. Although the prediction of ccRCC prognosis is primarily based on imaging and tumor-node-metastasis (TNM) staging, no other reliable prognostic tools are available for clinical use 5. Thus, more effective biomarkers are necessary to obtain an early diagnosis and better treatment options for ccRCC.

Lymphangiogenesis refers to the development of lymphatic vascularization during inflammation, wound healing, tumor progression, and tumor metastasis 6. Studies have confirmed that lymphangiogenesis is intimately associated with lymphatic metastasis, distant metastasis, and adverse clinical outcomes in a variety of tumor types, including ccRCC 7-12. Moreover, lymphatic vessels also represent an essential component of the immune microenvironment. It has been reported that lymphangiogenesis can regulate the trafficking and viability of immune cell populations and affect immunotherapy efficacy 13-15. Lymphangiogenesis is a complex process regulated by a number of factors and triggered by many genes, and lymphangiogenesis-related genes (LRGs) may represent prognostic genes in human tumors. The current study suggested that prospero homeobox 1 (PROX 1) and vascular endothelial growth factor C (VEGF-C) may play an important role in lymphangiogenesis and function as promoters of lymphatic metastasis 16. In addition, various molecular markers have been found to be correlated with lymphangiogenesis, including angiopoietin (ANG), fibroblast growth factor2 (FGF2), hepatocyte growth factor (HGF), and IL-17 17. Lymphangiogenic factors (e.g., VEGF-C and VEGF-D) can induce the metastatic spread of tumors in mouse models of cancer 18. Several studies have suggested that certain LRGs are associated with the development and prognosis of ccRCC, including VEGF-C, vascular endothelial growth factor D (VEGF-D), and lymphatic vessel endothelial hyaluronan receptor 1 (LYVE1) 19-21. However, no studies have investigated whether LRG can predict tumor progression and prognosis.

We screened the LRG gene set, constructed a reliable LRG model to assess the outcomes of ccRCC, and evaluated the relationship between the LRG signature and immune microenvironment. The predictive ability of the LRG signature was verified in validation cohorts. A function enrichment analysis revealed that LRGs were involved in the processes of tumor progression, tumor metastasis, tumor immunity and drug sensitivity. We further elucidated an association between the LRG signature and clinical profiles, immune features, somatic mutation, and drug sensitivity. Both immunohistochemistry (IHC) and immunofluorescence staining were performed to validate the relationship between lymphangiogenesis and immunity in tumor sections of ccRCC patients. Overall, our data suggests that LRG signature can effectively predict ccRCC prognosis, and the LRG signature is associated with an immunosuppressive phenotype. Therefore, this LRG signature can be used as a target for the precise treatment of ccRCC patients and provide support for assessing the efficacy of immunotherapy.

## Methods

### Data Collection

**Figure 1:**
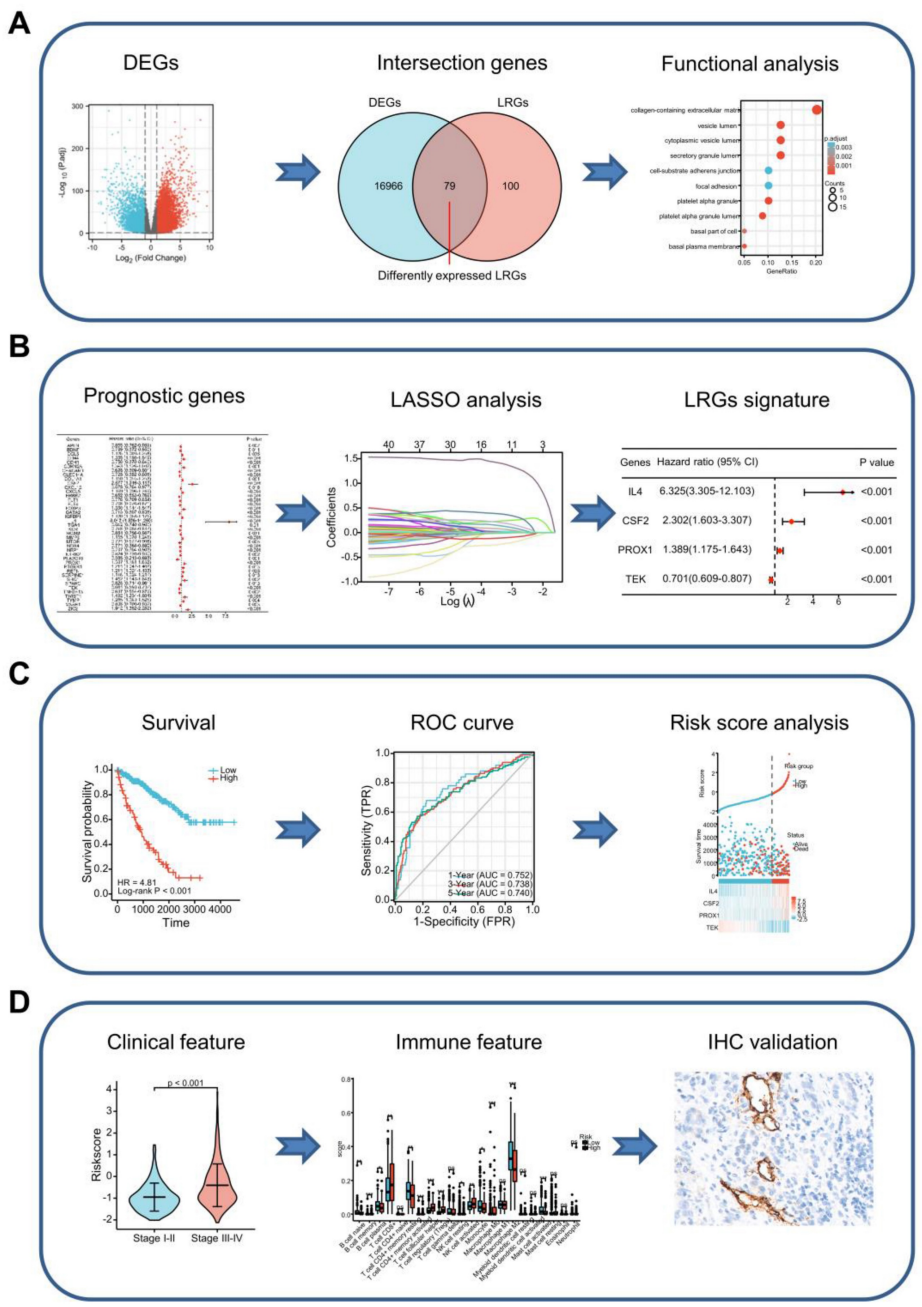
Graph showing the processes used in this study. **(A)** Screening of differentially expressed LRGs and subsequent functional enrichment analysis. **(B)** Construction of the LRG signature. **(C)** Validation of the efficacy and accuracy of the LRG signature using a variety of methods in different datasets. **(D)** Analysis of the LRG signature predicting clinical features and immune features. The relationship between the LRG signature and immune features as verified by IHC and immunofluorescence staining in our cohort of patients. LRG, lymphangiogenesis-related gene; IHC, immunohistochemistry.

Figure 1 presents a flow chart depicting the process used to analyze data in our study. Bulk RNA-seq data were obtained from The Cancer Genome Atlas (TCGA) database, including 530 ccRCC specimens and 72 paracancerous specimens. The bulk RNA-seq data of external validation cohort contained 91 ccRCC specimens was accessed from the International Cancer Genome Consortium (ICGC) database. The level of gene expression was measured as the number of transcripts per million reads (TPM). The baseline data of the enrolled patients are outlined in Supplementary Table 1. The 179 LRGs were sourced from the NCBI-Gene database using the keyword “lymphangiogenesis” and from the MSigDB database, using “GOBP_LYMPHANGIOGENESIS, GOBP_REGULATION_OF_LYMPHANGIO GENESIS and PID_LYMPH_ ANGIOGENESIS_PATHWAY”. The LRG set is presented in Supplementary Table 2.

### Selecting Differentially Expressed Lymphangiogenesis-related Genes and Functional Enrichment Analysis

The “Deseq2” package was applied to identify the differentially expressed genes (DEGs) among ccRCC and paracancerous specimens in the TCGA dataset. The criteria for DEGs consisted of Gene Expression alteration with |Log2Fold Change|≥1. The differentially expressed LRGs between DEGs and LRGs were analyzed using the "Venn" package. A functional enrichment analysis was performed using the R package "Clusterprofiler".

### Construction and Validation of the LRG Prognostic Signature

Patients in the TCGA dataset were divided into training (n=353) and validation (n=177) cohorts to construct and validate the LRG signature. A univariate Cox analysis was used to select prognostic genes with a threshold of *P* < 0.1. Further selection of prognostic genes was performed using least absolute shrinkage and selection operator (LASSO) analysis and multivariate Cox analysis in the training cohort. The LRG model was constructed using candidate LRGs and their regression coefficients.

ccRCC patients were divided into high-risk and low-risk groups according to the optimal cut-off values of risk scores gained from the R package "surv_cutpoint". We performed PCA analysis and t-SNE analysis using the "Rtsne" and "ggplot2" R packages to discriminate the distribution of individuals belonging to different risk groups. A Kaplan-Meier Analysis and receiver operating characteristic (ROC) curve were utilized to detect the effectiveness of the LRG signature. Identical risk score algorithms and cut-off points were used in the TCGA validation cohort, the TCGA total cohort, and the external ICGC validation cohort to verify the LRG signature. The LRG signature is displayed as a risk map.

### Tumor Microenvironment and Immune Status Analysis

The tumor stemness index based on mRNA expression (mRNAsi) was performed on TCGA tumor samples using the assessment algorithm described by Malta et al. 22. The immune and stromal scores were calculated using "estimates" package. We performed an infiltration score of immune cells using different algorithms, including QUANTISEQ, XCELL, and EPIC. In addition, the association between risk scores and immune regulatory genes was investigated in the TCGA dataset.

### Drug Sensitivity Analysis

The treatment response was predicted for individual samples obtained from the accessible pharmaceutical genomics database [the Genomics of Drug Sensitivity in Cancer (GDSC), https://www.cancerrxgene.org]. The half-maximal inhibitory concentration (IC50) of each sample was assessed using R package "pRRophetic".

### Evaluation of IHC and Immunofluorescence Staining

IHC analysis was performed on formalin-fixed and paraffin-embedded samples 23. Briefly, after dewaxing, rehydrating, antigen retrieval, inactivating endogenous peroxidase, and blocking non-specific binding, 5 μm-thick sections were incubated with the anti-D2-40 (1:500, ZSGB-BIO, Beijing, China), anti-CD8 (1:500, Thermo Fisher Scientific, Waltham, MA, USA), and anti-CD163 (1:1000, ZSGB-BIO, Beijing, China) overnight at 4℃. Subsequently, the sections were incubated with the corresponding secondary antibody and stained with the EnVision Detection System (DAKO).

Multiplex immunofluorescence was detected using a PANO multiplexed immunofluorescence kit (Cat#0079100020, PANOVUE, Beijing, China) in accordance with the manufacturer's protocol 24. Briefly, the slices were de-paraffinized and hydrated. Microwave treatment was applied for antigen retrieval. After a 10-min blocking step, the sections were incubated overnight at 4°C with the primary antibody. The sections were subsequently incubated with the appropriate secondary antibodies for 10 min at room temperature. Finally, one of the three tyramide signal amplification fluorophores (Opal 520, 570, and 690) was applied to the sections for 10 min. This sequence of steps (except deparaffinization and hydration) was repeated, beginning with blocking and terminating with the microwave treatment. The following primary antibodies used for the multiplex immunofluorescence staining: anti-CD8 (1:1000, Thermo Fisher Scientific, Waltham, MA, USA), anti-PD1 (1:1000, Cell Signaling Technology, Beverly, MA, USA), and anti-LAG3 (1:1000, Cell Signaling Technology, Beverly, MA, USA). Finally, the slides were scanned using a Vectra Polaris^TM^ Automated Quantitative Pathology Imaging System (PerkinElmer, MA, USA).

The proliferation of lymphatic endothelial cells is a prerequisite for lymphangiogenesis 13. We analyzed the relationship between D2-40^+^ lymphatic vessel density (LVD) and immune cell infiltration by IHC and immunofluorescence staining. D2-40^+^ LVD was defined as the number of vessels per mm^2^. D2-40^+^ LVD, CD8^+^ tumor-infiltrating lymphocytes (TILs), and CD163^+^ tumor-associated macrophages (TAMs) were evaluated by images captured at high-power representative fields (×200 magnification) and counted manually. Immunofluorescence staining was performed for CD8, PD-1, and LAG3. CD8^+^ PD-1^+^ and CD8^+^ LAG3^+^ TILs were evaluated by obtaining images at high-power representative fields (×200 magnification) and counted manually. IHC and immunofluorescence staining were performed on the serial tumor sections of 15 ccRCC patients. The local ccRCC cohort characteristics are presented in Supplementary Table 3. Forty-eight zones were randomly defined and quantified for these ccRCC patients to analyze the correlation between D2-40^+^ LVD and CD163^+^ TAMs, CD8^+^ TILs, CD8^+^ PD-1^+^ TILs and CD8^+^ LAG3^+^ TIL infiltration. Data are presented as the mean ± standard error to show the number of cells per mm^2^.

### Single-cell RNA sequencing data analysis

We have verified the selected genes (IL4, CSF2, PROX1 and TEK) expression in a public single-cell sequencing database and used bubble maps to show the results. The data Number we used was SRZ190804, and the data was downloaded from https://trace.ncbi.nlm.nih.gov/Traces/sra/sra.cgi?analysis=SRZ190804. The single-cell transcriptome data were analyzed using the “Seurat” package.

### Statistical analysis

R software (version 4.2.0) was used to perform the statistical analyses. Data were presented as the mean ± standard error, and *P* < 0.05 was considered statistically significant.

## Results

### Identification of Differentially Expressed LRGs and Functional Enrichment Analysis

**Figure 2:**
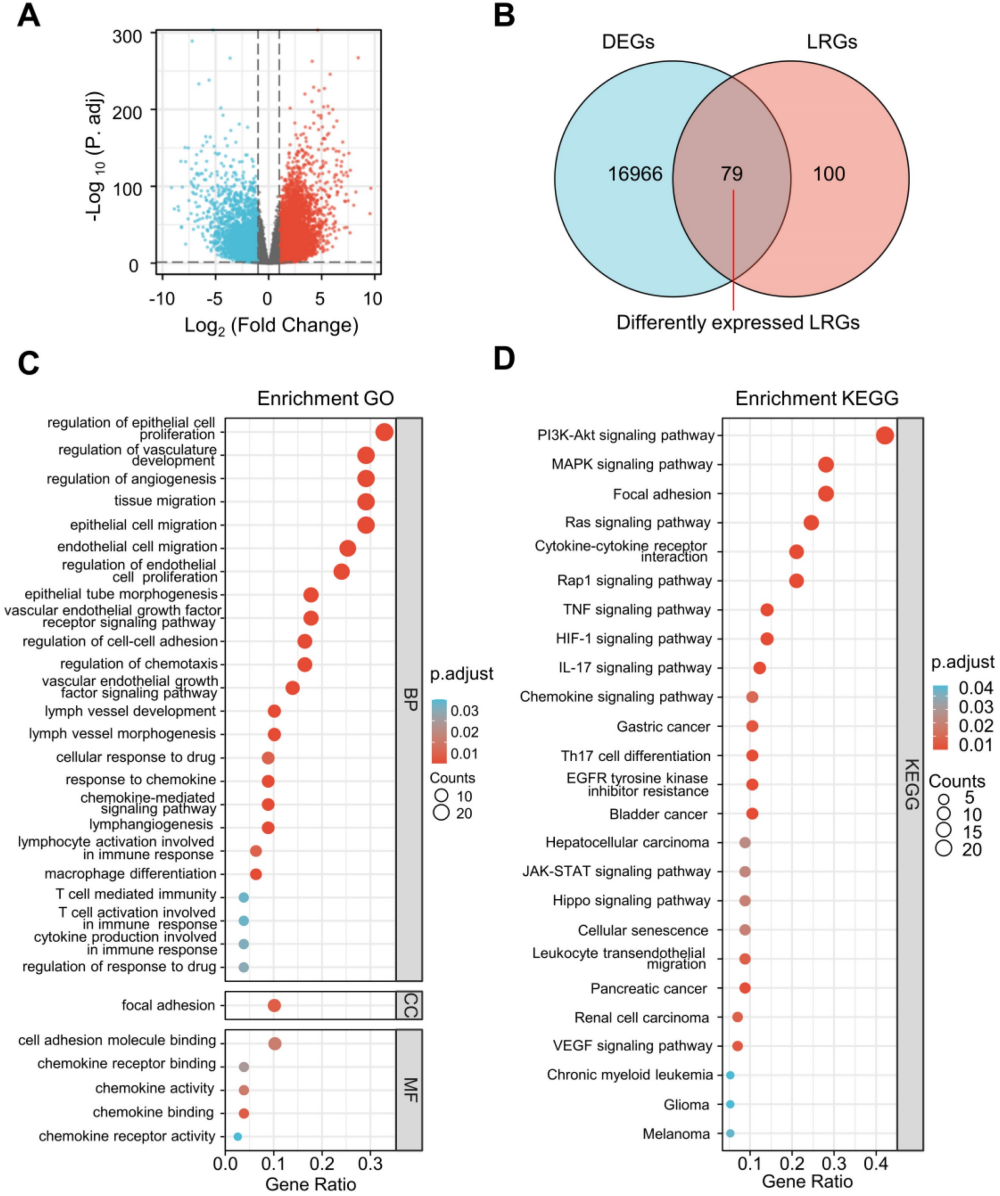
Selection of differentially expressed LRGs and functional enrichment analysis. **(A)** Volcano map showing the DEGs in the TCGA database (tumor vs. normal). **(B)** Venn gram showing 79 differentially expressed LRGs. **(C, D)** Bubble map of significantly enriched GO terms **(A)** and KEGG pathways **(B)** in the TCGA dataset. LRGs, lymphangiogenesis-related genes; DEGs, differentially expressed genes.

The differential expression analysis was performed between ccRCC specimens (n = 530) and paracancerous specimens (n = 72) from the TCGA cohort. A total of 17,226 DEGs were identified (Figure 2A) and 79 differentially expressed LRGs were found at the intersection of DEGs and LRGs (Figure 2B).

Remarkable GO and KEGG enrichment terms were displayed using bubble plots (Figure 2C and D). Several lymphangiogenesis-related molecular functions were enriched, including lymphangiogenesis, lymph vessel development, vascular endothelial growth factor receptor signaling pathway, vascular endothelial growth factor signaling pathway, and lymph vessel morphogenesis (Figure 2C). The differentially expressed LRGs were primarily enriched in the biological processes, including regulation of cellular proliferation, migration, and adhesion, as well as the humoral immune response, T cell activation, macrophage differentiation, chemokine-mediated signaling pathway, and cellular response to drugs (Figure 2C). Analysis of the KEGG pathway revealed that differentially expressed LRGs were significantly correlated with cancer-related pathways, including the PI3K-Akt signaling pathway, MAPK signaling pathway, Ras signaling pathway, focal adhesion, JAK-STAT signaling pathway, renal cell carcinoma, and VEGF signaling pathway (Figure 2D).

### Construction of the LRG Signature

**Figure 3:**
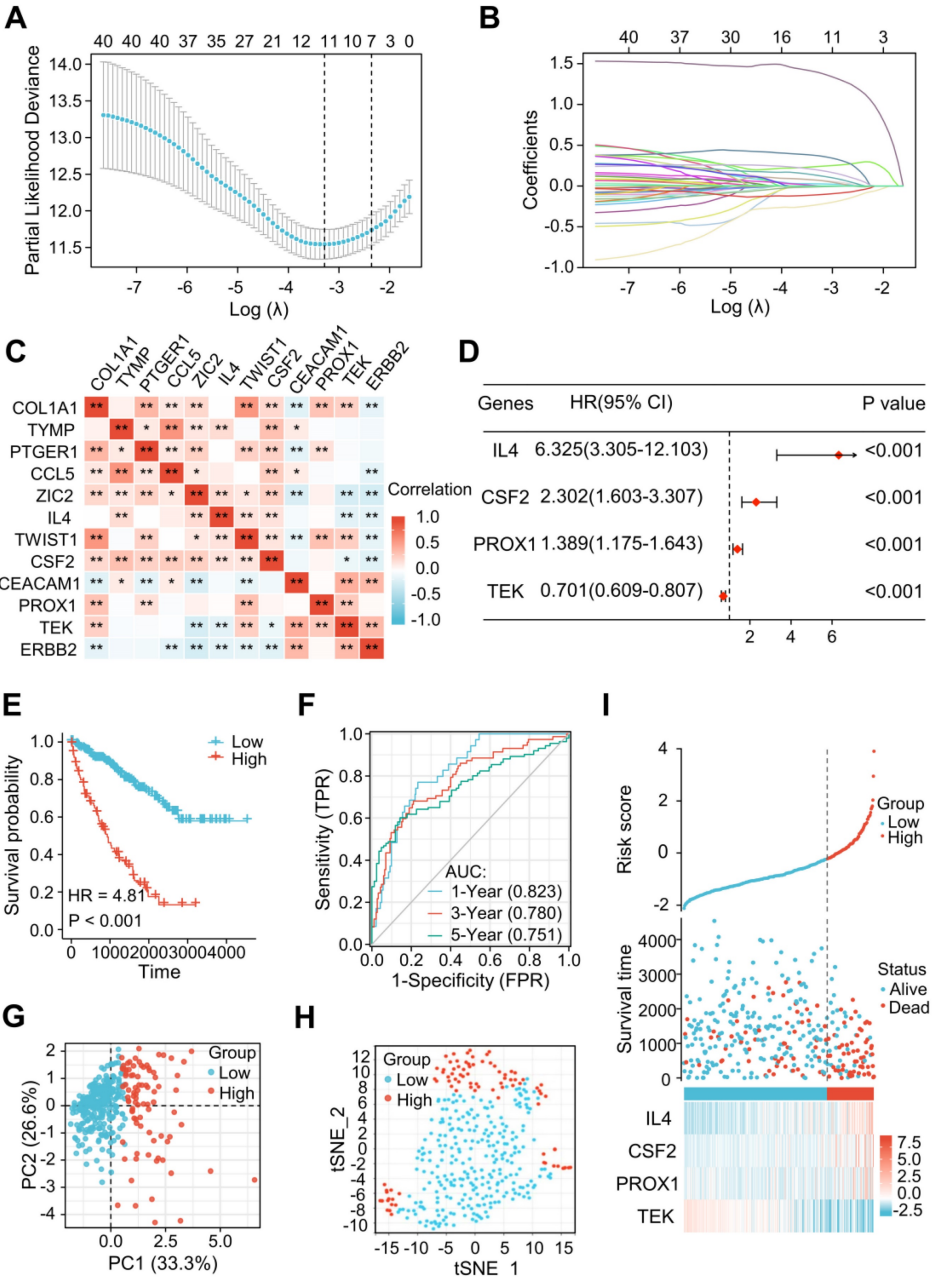
A novel signature of the LRGs constructed in the TCGA training cohort. **(A, B)** There were 12 genes selected by a LASSO analysis. **(C)** The heatmap showed a correlation between the 12 genes. **(D)** Construction of a 4-gene LRG signature using a multivariate Cox analysis. **(E)** Survival curves of the high- and low-risk group. **(F)** 1, 3, and 5-year time-dependent ROC curves. **(G)** PCA plot in high- and low-risk groups. **(H)** Analysis of t-SNE among different groups. **(I)** Risk factor association diagram. LRGs, lymphangiogenesis-related genes; ROC, receiver operating characteristic; PCA, Principal Component Analysis; *P < 0.05; **P < 0.01.

Among the 79 differentially expressed LRGs, 40 LRGs were identified by a univariate Cox analysis in the train cohort of TCGA dataset (n=353) (Figure S1). There were 12 genes identified by a LASSO analysis using the minimum lambda value (Figure 3A and B). Figure 3C shows the correlation among these 12 genes. The multivariate Cox analysis of these 12 genes revealed 4 significantly prognostic genes, including interleukin-4 (IL-4), colony stimulating factor 2 (CSF2), prospero homeobox 1 (PROX1), and TEK receptor Tyrosine Kinase (TEK). (Figure 3D, *P* < 0.05). Figure S2 shows the differential levels of expression of these 4 genes between ccRCC and normal tissue. These 4 genes could be further classified into protective (TEK) and risky genes (IL4, CSF2, and PROX1) according to the hazard ratio (HR) (Figure 3D). Risk score = (1.84447 × IL4 expression) + (0.83394 × CSF2 expression) + (0.32870 × PROX1 expression) + (-0.35554 × TEK expression). The sample was then divided into low-risk and high-risk groups in accordance with the optimal cut-off point (-0.22103). A Kaplan-Meier survival analysis indicated a shorter OS in the high-risk group (HR = 4.81 [2.97-7.78], *P* < 0.001) (Figure 3E). The AUCs indicated good predictive capability of the risk scores in terms of OS rates at 1, 3, and 5 years (0.823, 0.780, and 0.751) (Figure 3F). The PCA and t-SNE maps showed a distinct dimension among different groups (Figures 3G and H). Risk map of the risk score distribution, survival status and expression landscape showed that the survival status was strongly related to the risk score (Figures 3I).

### Validation of the Prognostic Value of the LRG Signature in Different ccRCC Cohorts

**Figure 4:**
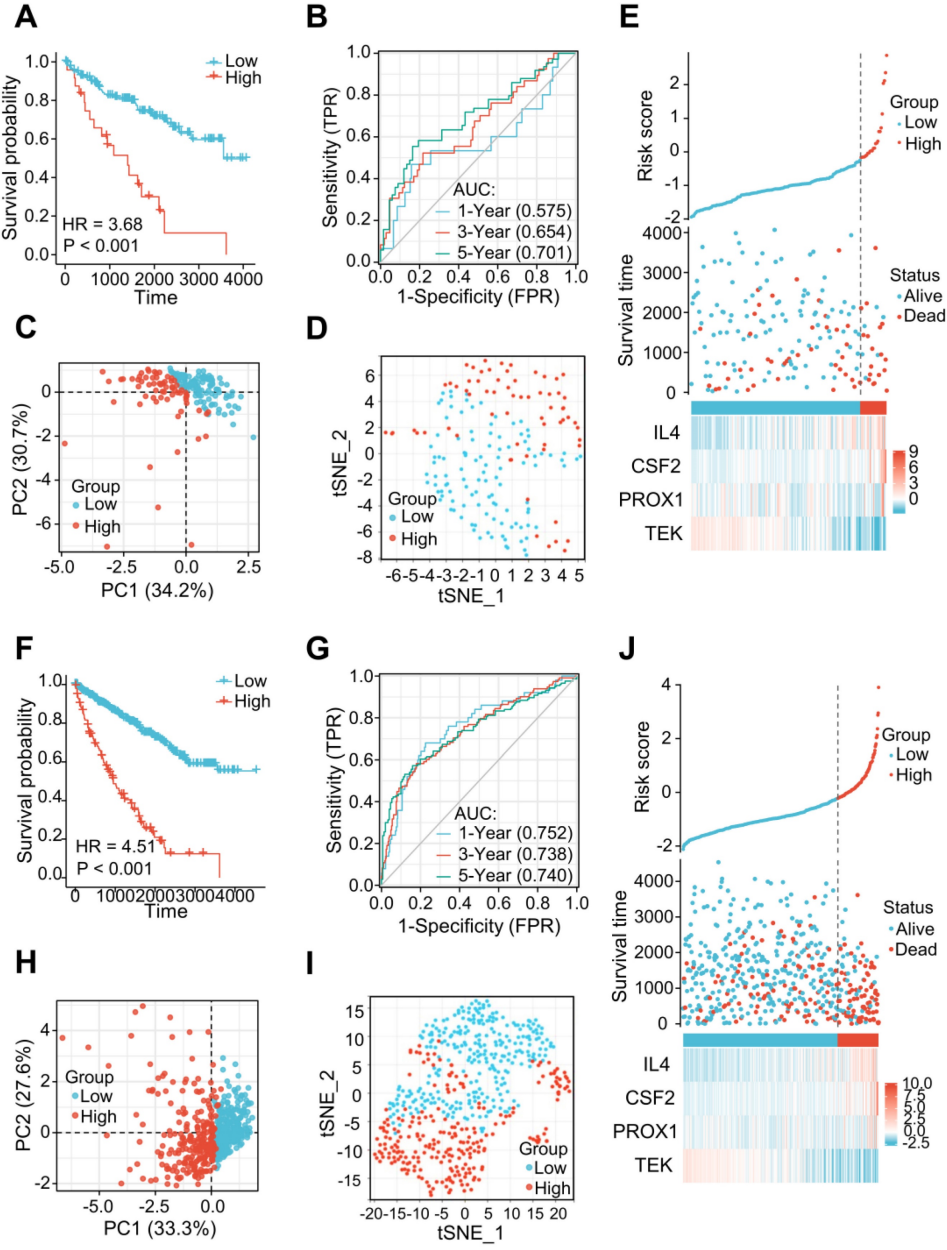
The LRG signatures were verified in the validation cohort and total cohort of the TCGA dataset. **(A, F)** Survival curve in different groups of validation cohort and total cohort. **(B, G)** 1, 3, and 5-year time-dependent ROC curves in the validation cohort and total cohort. **(C, H)** PCA plot in high- and low-risk groups of the validation cohort and total cohort. **(D, I)** Analysis of the t-SNE among different groups in the validation cohort and total cohort. **(E, J)** Association diagrams of risk factors in the validation and total cohorts. LRG, lymphangiogenesis-related gene; ROC, receiver operating characteristic; PCA, Principal Component Analysis.

The 177 ccRCC samples in the TCGA dataset were clustered as an internal validation cohort using the same cut-off values to verify the appropriateness and stability of the LRG signature. In accordance with the results above, patients with high-risk score have poorer OS (HR= 3.68 (1.61-8.42), *P* < 0.001) (Figure 4A). The AUCs for the 1-, 3-, and 5-year OS rates were 0.575, 0.654, and 0.701, respectively (Figure 4B). PCA and t-SNE analyses also revealed varying dimensionality in the different groups (Figure 4C and D). A risk map of the risk score distribution, survival status, and expression landscape showed that survival status was strongly related to the risk score (Figures 4E). A comparative analysis was also conducted for the total cohort (n = 530) of the TCGA dataset, which reinforced the value of risk scores to predict the prognosis (Figures 4F-J). The AUCs for the 1-, 3-, and 5-year OS rates were 0.752, 0.738, and 0.748, respectively in the total cohort (Figures 4G).

**Figure 5:**
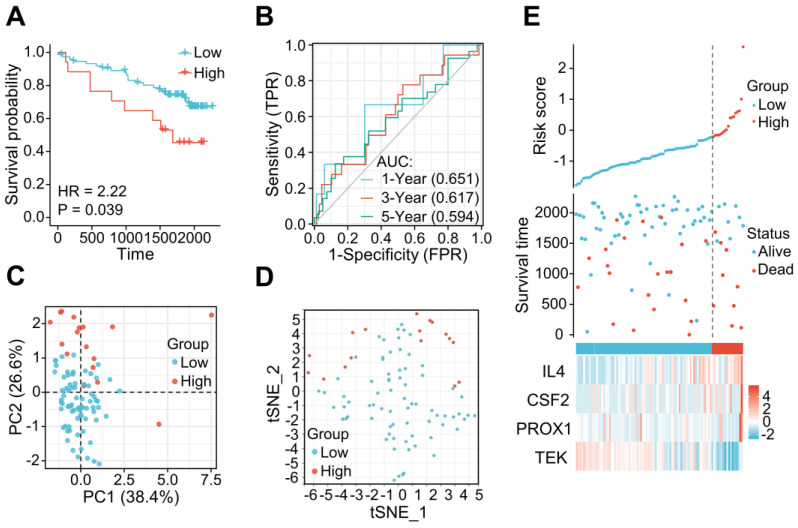
The LRG signature was verified in the external ICGC dataset. **(A)** Survival curve in each of the different groups. **(B)** 1, 3, and 5-year time-dependent ROC curves. **(C)** PCA plot in the high- and low-risk groups. **(D)** Analysis of t-SNE among high- and low-risk groups. **(E)** Risk factor association diagram. LRG, lymphangiogenesis-related gene; ROC, receiver operating characteristic.

The applicability and stability of the LRG signature was also validated in an external ICGC cohort containing 91 ccRCC samples. A Kaplan-Meier survival analysis revealed a shorter OS in the high-risk group (HR = 2.22 (0.84 - 5.87), *P* = 0.039) (Figure 5A). The AUCs indicated good predictive capability of the risk scores in terms of the OS rates at 1, 3, and 5 years (0.651, 0.617, and 0.594, respectively) (Figure 5B). Analyses of PCA and t-SNE also revealed variable dimensionality (Figure 5C and D). A risk map of the risk score distribution, survival status, and expression landscape suggested that the survival status was strongly related to the risk score (Figures 5E).

### Clinical Relevance of the LRG Signature

**Figure 6:**
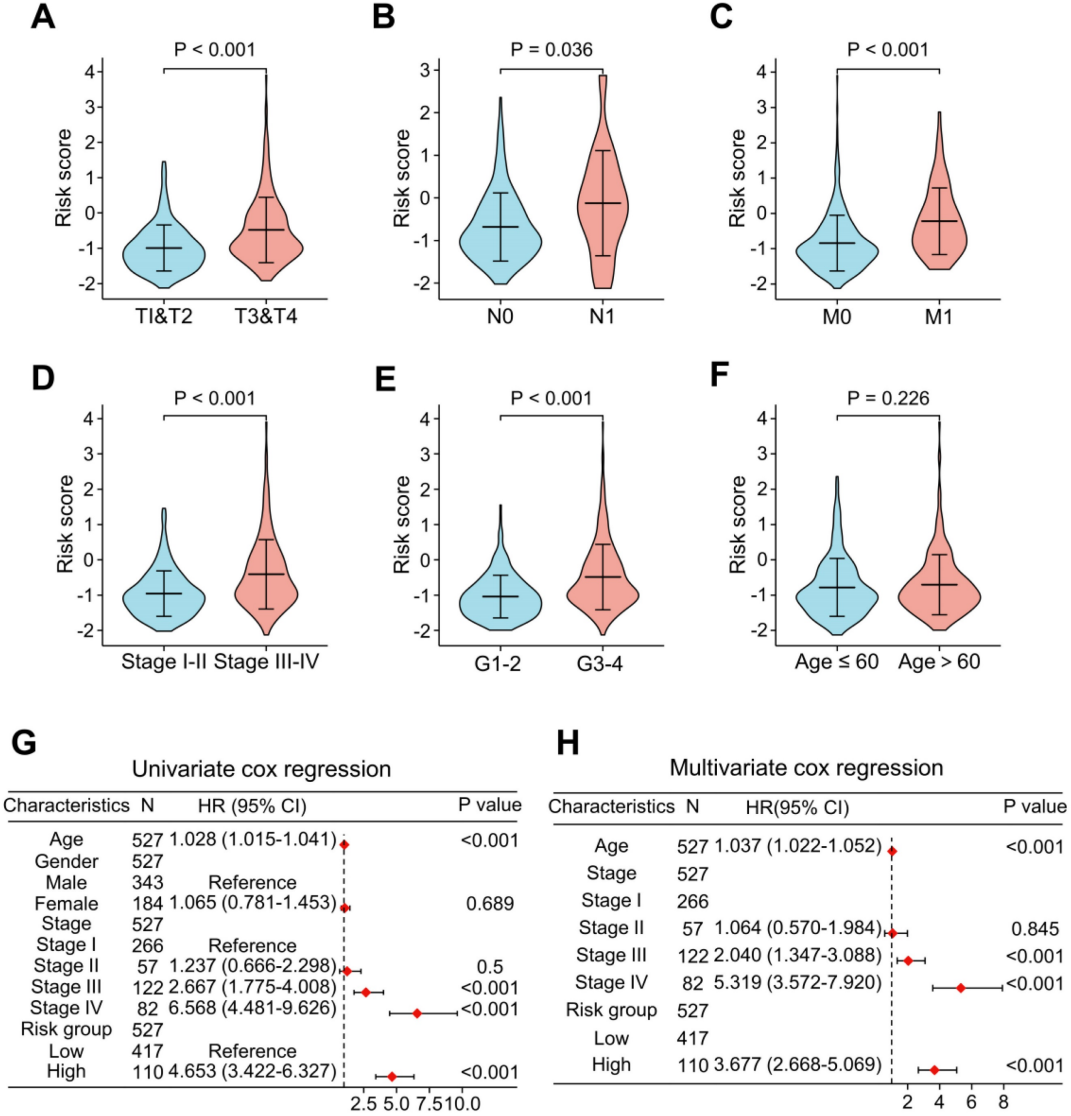
Analysis of the relevance of clinical features and independent prognostic value of the LRG signature. Correlation analysis of the risk scores with clinicopathological features of: T stage **(A)**, N stage **(B)**, M stage **(C)**, pathological stage **(D)**, histological grading **(E)**, and age **(F)** in the TCGA dataset. Forest plots showing the risk scores as independent prognostic factors for overall survival of ccRCC patients using univariate Cox analysis **(G)** and multivariate Cox analysis **(H)**. LRG, lymphangiogenesis-related gene.

To add value to the clinical application of the LRG signature, we analyzed the correlation between risk scores and clinical features in the TCGA cohort (Figure 6). The results showed that the advanced T stages, N stages, M stages, pathologic stage, and histologic grade had a significantly elevated risk score (Figure 6A-E). However, there was no meaningful association between age and risk score (Figure 6F). Univariate and multivariate Cox analyses revealed that age, pathological stage, and risk score were independent predictors of OS (Figure 6G and H).

### Immunological Features and Tumor Microenvironment Analysis of the LRG Signature

**Figure 7:**
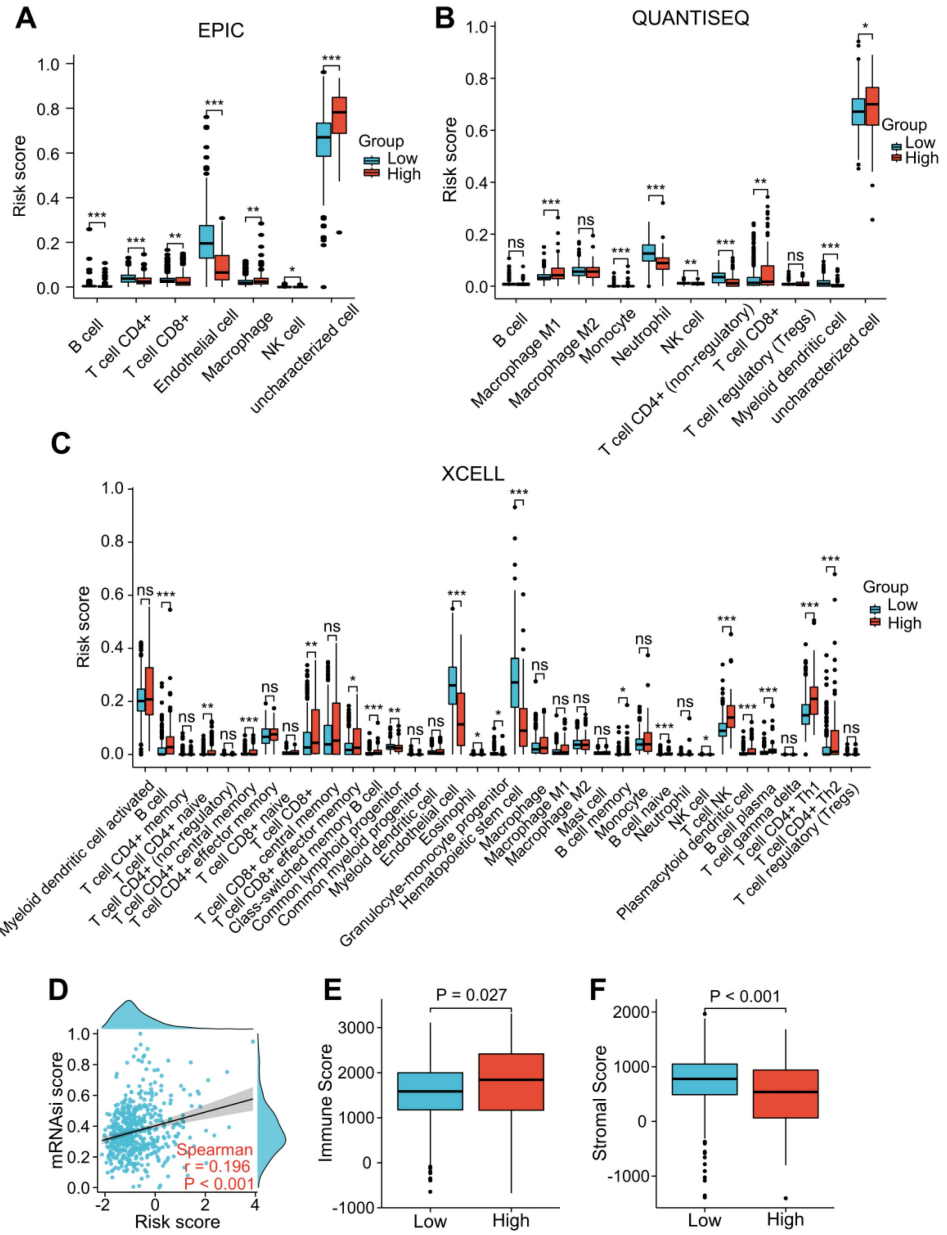
Correlation analysis of the LRG signature and immunity. **(A-C)** Analysis of the difference in the level of immune cell infiltration among different groups using “EPIC” **(A)**, “QUANTISEQ” **(B)**, and “Xcell” **(C)** algorithms. **(D)** Association between the LRG signature and tumor stemness index (mRNAsi). **(E, F)** Differences in the immune score **(E)** and stromal score **(F)**. LRG, lymphangiogenesis-related gene; *P < 0.05; **P < 0.01; ***P < 0.001.

“EPIC”, “XCELL”, and “QUANTISEQ” were performed to assess the level of immune cell infiltration among the different groups. The EPIC results showed that the CD8^+^ T cell and macrophage scores were significantly higher in the patients with a higher risk score (Figure 7A). Similar results were found in the QUANTISEQ analysis, which showed that higher risk score patients had higher scores of CD8^+^ T cells and M1 macrophages (Figure 7B). The XCELL results showed that higher risk score patients had a higher score of B cells, Th2 cells, and CD8^+^ T cells (Figure 7C). We integrated tumor stemness score, stromal score, and immune score to analyze the interaction of each of these scores with risk scores. Tumor stemness was measured by mRNAsi. Our findings indicated that increased risk scores were significantly correlated with improved mRNAsi (r = 0.196, *P* < 0.001; Figure 7D). In addition, the ESTIMATE results indicated that patients with higher risk scores had lower stromal scores and higher immune scores than those with lower risk scores (Figure 7E and F), suggesting that LRG signature may be correlated with the TME in ccRCC patients.

**Figure 8:**
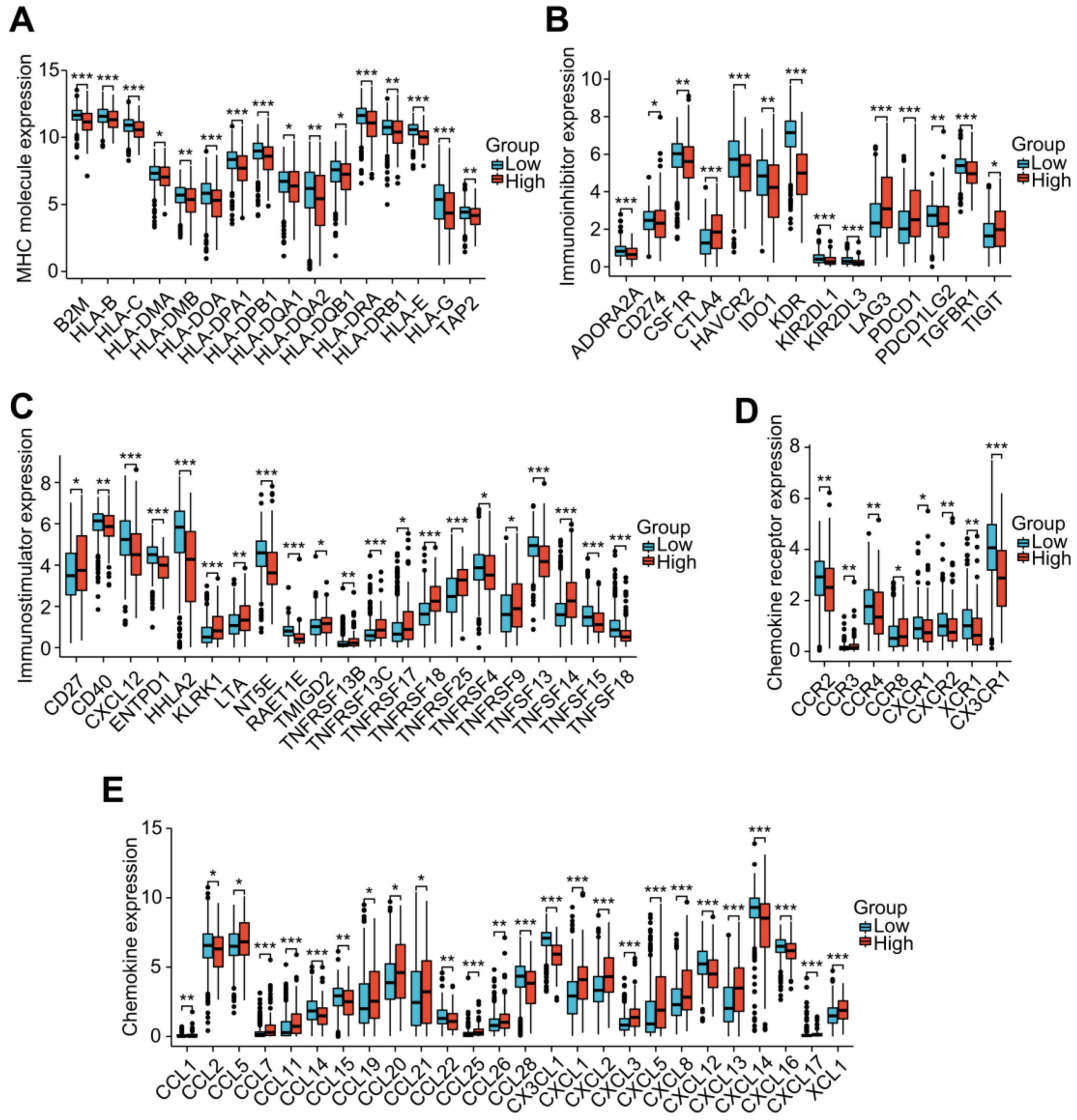
Correlation analysis of the LRG signature and immunoregulatory genes. Box plots displaying the immunomodulatory genes exhibiting significantly different expression between the high-risk and low-risk groups, including **(A)** MHC genes, **(B)** immunosuppressive genes, **(C)** immune activation genes, **(D)** chemokine receptors, and **(E)** chemokines. LRG, lymphangiogenesis-related gene; MHC, major histocompatibility complex; *P < 0.05; **P < 0.01; ***P < 0.001.

To investigate the role of the LRG features in immunoregulation, we further analyzed the differences in the expression of immunoregulatory genes related to MHC molecules, immunosuppression, immune activation, chemokine receptors, and chemokines among different groups (Figure 8A-E). Our outcomes indicated that the expression of these immunomodulatory genes significantly differed among distant groups. Among the immunoregulatory genes related to T cell exhaustion markers, most genes displayed higher expression in patients with higher risk scores. Patients in the high-risk group had higher expression of common T cell exhaustion markers, including PD-1, LAG3, CTLA-4, and TIGIT (Figure 8A).

### Validation of the Correlation between Lymphangiogenesis and Immune Cells by IHC and Immunofluorescence Staining

**Figure 9:**
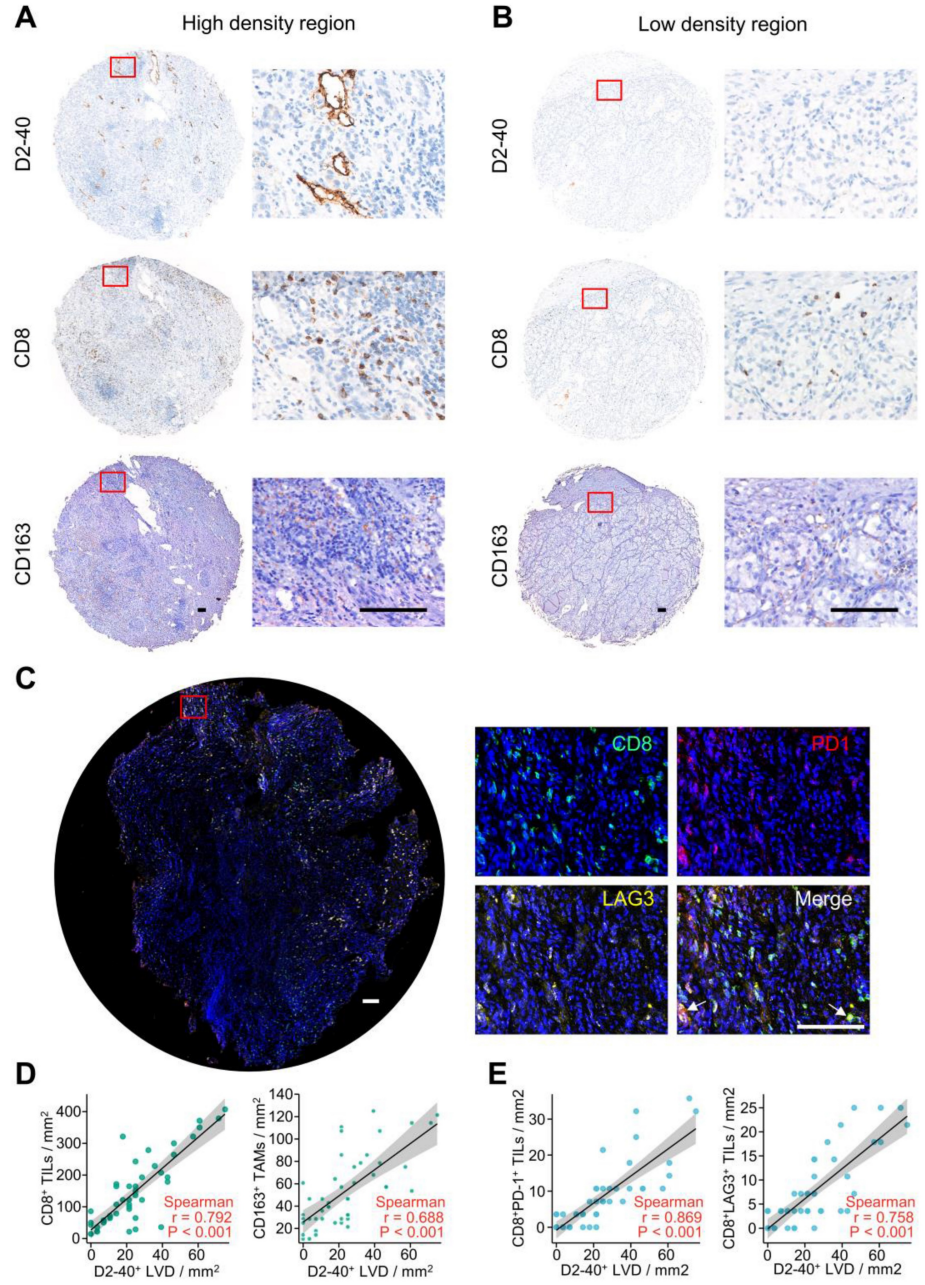
Lymphangiogenesis in immunosuppressive tumor tissues. **(A, B)** Representative images of the D2-40^+^ LVD with CD8^+^ TILs or CD163^+^ TAM infiltration from patients with increased D2-40^+^ LVD **(A)** and those with decreased D2-40^+^ LVD **(B)**. **(C)** Representative images of three-color immunofluorescence staining for CD8 (green), PD-1 (red), and LAG3 (yellow) with 4ʹ,6-diamidino-2-phenylindole counterstaining (DAPI) (blue). **(D)** Association between D2-40^+^ LVD and CD8^+^ TILs or CD163^+^ TAMs infiltration in ccRCC tissues. **(E)** Association of D2-40^+^ LVD and CD8^+^ PD-1^+^ TIL or CD8^+^ LAG3^+^ TIL infiltration in the ccRCC tissues. LVD, lymphatic vessel density; TILs, tumor-infiltrating lymphocytes; TAM, tumor-associated macrophage. Scale bars indicate 100 μm.

To verify the interaction between lymphangiogenesis and immunocyte infiltration, IHC staining was used to examine the correlation of D2-40^+^ LVD and the density of CD8^+^ TILs and CD163^+^ TAMs in the ccRCC samples. Representative images of the D2-40^+^ lymphatic vessel, CD8^+^ TILs, and CD163^+^ TAMs in the high- and low-density regions are shown in Figure 9A and B. CD8^+^ TILs and CD163^+^ TAMs were enriched in the high-density regions of the D2-40^+^ lymphatic vessel (Figure 9A), whereas CD8^+^ TILs and CD163^+^ TAMs were rarely observed in the low-density regions of the D2-40^+^ lymphatic vessel (Figure 9B). The results of the scatter plots showed a positive correlation for D2-40^+^ LVD and the density of CD8^+^ TILs, or CD163^+^ TAMs in the ccRCC tissues (Figure 9D). Since the LRG signature was correlated with T cell exhaustion markers in ccRCC patients, we further investigated the association between D2-40^+^ LVD with exhausted T cells in the ccRCC tissues by immunofluorescence staining for CD8, PD-1, and LAG3. The immunofluorescence staining revealed that the D2-40^+^ lymphatic vessel and CD8^+^ PD-1^+^ TILs/CD8^+^ LAG3^+^ TILs were enriched in the same regions (Figure 9C). Increased D2-40^+^ LVD was positively associated with a high density of CD8^+^ PD1^+^ TILs or CD8^+^ LAG3^+^ TILs (Figure 9E). These results indicate that lymphangiogenesis was correlated with immune cell infiltration and a T cell exhaustion phenotype in ccRCC patients.

### Tumor Somatic Mutation Analysis

**Figure 10:**
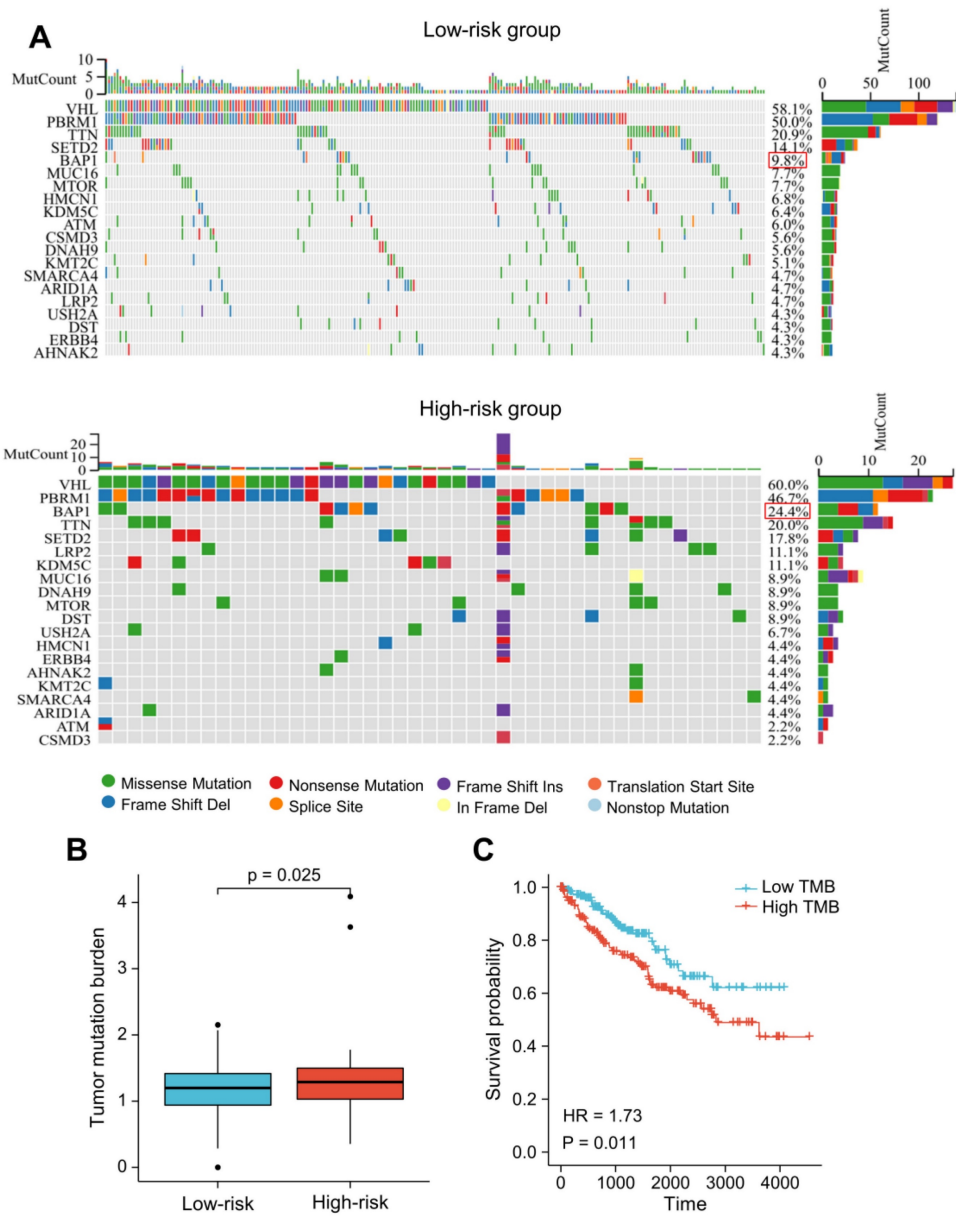
Somatic mutation analysis based on the LRG signature. **(A)** Analysis of the 20 genes with the greatest number of mutations in the high and low-risk groups. **(B)** Differences in the presence of the tumor mutation burden (TMB) between the high- and low-risk groups. **(C)** Analysis of overall survival of patients with different TMB. LRG, lymphangiogenesis-related gene; TMB, tumor mutation burden.

We utilized R package "maftools" to identify the differences between the high- and low-risk groups regarding the tumor mutation burden (TMB). The nucleotide variation information from the TCGA dataset was used to obtain the mutation profile for each sample. In ccRCC patients, the top 20 genes with the highest mutation rates were: VHL, PBRM1, TTN, SETD2, BAP1, MUC16, MTOR, KDM5C, HMCN1, LRP2, DNAH9, ATM, CSMD3, ARID1A, KMT2C, DST, USH2A, SMARCA4, ERBB4, AHNAK2 (Figure 10A). The majority of mutations were missense mutations, with a higher rate of BAP1 mutations in patients with higher risk scores (24.4% vs. 9.8%, *P* < 0.05; Figure 10A). The TMB score was significantly higher in patients with higher risk scores (Figure 10B). Additionally, the prognosis of ccRCC patients was poor as indicated by high TMB (HR = 1.73 (1.14-2.64); *P* = 0.011) (Figure 10C).

### Association Between the LRG Signature and Drug Sensitivity

**Figure 11:**
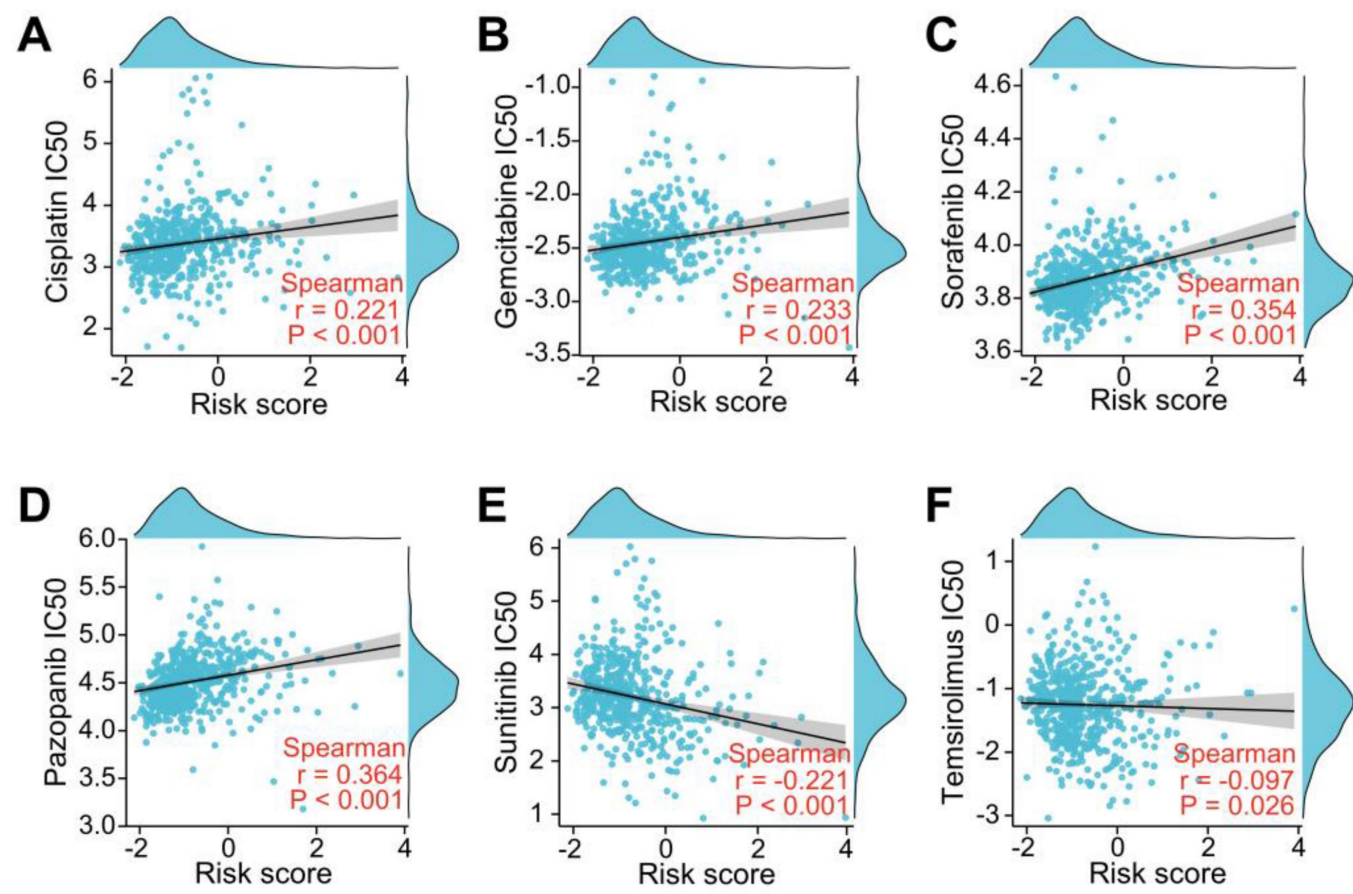
Evaluation of drug sensitivity based on the LRG signature. Scatter plots showing the correlation between the LRG signature and IC50 value of cisplatin **(A)**, gemcitabine **(B)**, sorafenib **(C)**, pazopanib **(D)**, sunitinib **(E)**, and temsirolimus **(F)** in the TCGA dataset. LRG, lymphangiogenesis-related gene.

To investigate whether the LRGs characteristics could predict sensitivity to common drug, we explored the relationship between the LRG signature and IC50 levels of several therapeutic agents, including pazopanib, sorafenib, sunitinib, temilomide, cisplatin, and gemcitabine. The scatter plot results indicated that an increased risk score was correlated with elevated IC50 levels of cisplatin, gemcitabine, sorafenib, and pazopanib (Figure 11A-D). Conversely, an elevated risk score was correlated with a decreased IC50 levels of sunitinib and temsirolimus (Figure 11E-F). These findings suggest that the risk score could be used as potential guidance for therapeutic drugs used for the clinical treatment of ccRCC patients.

### Exploration of the Signature Genes in a Single-Cell Level

The data include single-celled sequencing data of 6 patients. Two of them, named UT1 and UT2, had not received any medical treatment before single-cell sequencing, which were selected for subsequent analyses. The TNM staging of these two patients was T3aN0M0 (UT1) and T4N1M1 (UT2). The main cell populations were identified according to corresponding markers (Figure S3A). The four genes in our signature were mainly expressed in stromal cells. In UT1 lymphatic metastasis did not occur. IL4, CSF2, and PROX1 were not expressed, while TEK was highly expressed in B cells and vascular endothelium cells (Figure S3B). In UT2, lymphatic metastasis occurred. TEK expression was inhibited, while IL4 was highly expressed in Tissue-resident T cells, and CSF2 was highly expressed in Tissue-resident T cells, and CD4+ Activated IEG T cells, and PROX1 was highly expressed in the ccRCC cell, renal epithelium cell, monocyte, and TAM (Figure S3C). The results are consistent with the conclusion of our paper, IL4, CSF2, and PROX1 are risky genes, and TEK is a protective gene.

## Discussion

Tumor-associated lymphangiogenesis is intimately involved in lymph node metastases, remote metastases, and adverse outcomes in a variety of tumor types, including ccRCC 7-12, 25. Furthermore, the lymphatic vascular system constitutes a vital part of the immediate environment of the immune system. Moreover, it serves as a critical artery by which the immune cells flow and play an active role in influencing the immunological response 26. The correlation between lymphangiogenesis and the tumor immune microenvironment/immunotherapeutic response has recently been described 27, 28. Although lymphangiogenesis is thought to play a crucial role in the tumor immune microenvironment and prognosis, little is known about the correlation between LRGs and progression/immunity in ccRCC patients. Moreover, the potential role of the LRG signature to serve as a potent therapy intervention for ccRCC remains poorly understood. Consequently, an investigation of how LRGs affect the progression and immunity of ccRCC is warranted, and there is a need to determine the potential prognostic value of LRGs in ccRCC patients.

A systematic analysis of the expression profiles of 179 ARGs in the TCGA and ICGC datasets combined with clinical data, was performed in this study. Differentially expressed LRGs were predominantly enriched in lymphangiogenesis and cancer-related pathways by enrichment analysis. Subsequently, a prognostic LRG signature integrating four LRGs was constructed and validated in this study. The LRG signature was found to be linked to several clinical features and could be used to independently assess the prognosis of ccRCC. We further explored the correlation between the LRG signature and TME, immunity, somatic mutation, and drug sensitivity. The IHC and immunofluorescence staining results validated the correlation between lymphangiogenesis with CD163^+^ TAMs, CD8^+^ TILs, exhausted CD8^+^PD-1^+^ T cells, and CD8^+^ LAG3^+^ T cells. This study is the first comprehensive assessment of the impact of the LRG signature in terms of clinical prognosis, immune characteristics, and drug sensitivity in ccRCC patients.

The novel LRG signature in our study was based on four genes (IL4, CSF2, PROX1, and TEK). IL-4 is a gene coding a pleiotropic cytokine, which has been suggested to be an essential cytokine for tissue repair, wound healing, and acute inflammation. In synergy with IL-4, B cell activating factor (BAFF) can drive the growth of lymphatic endothelial cells by stimulating B cell generation of lymphangiogenic elements VEGF-A and VEGF-C 29. Additionally, IL-4 can directly impact tumor proliferation and indirectly polarize TAMs to an M2-biased phenotype, thereby promoting tumorigenesis and growth 30. CSF2 is thought to be closely associated with macrophage production, differentiation, and phenotypic alterations 31. Additionally, it has been shown that adverse clinical outcomes are caused by CSF2 overexpression in bladder cancer 32. PROX1 plays a crucial role in the formation of the lymphatic vasculature. PROX1 is also a key marker on lymphatic endothelial cells and is thought to act in lymphatic endothelial cell sprouting and differentiation 33. It has been demonstrated that PROX1 promotes the growth, migration, and aggression of osteosarcoma cells *in vitro* 34. TEK is involved in stabilizing blood vessels and promoting vascular maturation 35. In addition, it has been discovered that TEK could predict the efficacy of immunotherapy and play a predictive role in the survival analysis of ccRCC patients 36. These findings suggested that the selected LRGs are strongly associated with lymphangiogenesis, tumor immune microenvironment, and prognosis in carcinomas, which provides a possibility of building a prognostic gene signature.

It has been considered that ccRCC is one of the most immunogenic cancers and the poor prognosis of ccRCC is closely related to its complex metabolic mechanism of progression, including the formation of an immunosuppressive microenvironment 37, 38. Our results showed that the proportion of CD8^+^ T cells, Th2 cells, and macrophages were obviously higher in patients with a higher risk score. It is generally accepted that the accumulation of CD8+ TILs inhibits tumor progression and is associated with a good clinical prognosis 27. Unlike the vast majority of cancers, ccRCC patients who have higher CD8^+^ TIL density are associated with a worse prognosis 39, 40. Previous studies have suggested that CD8^+^ TILs can possibly display an exhausted phenotype in ccRCC patients due to the expression of T cell exhaustion markers, such as PD-1, CTLA-4, LAG3, and TNFRSF9 41, 42. Furthermore, high CD8^+^ TIL infiltration is correlated with an immunosuppressive subset of immune cells (e.g., macrophages and Th2 cells) in ccRCC patients 43, 44. Therefore, upregulated T cell exhaustion markers, including TNFRSF9, PD-1, CTLA4, LAG3, and TIGIT, and the increased immunosuppressive subset of TAMs and Th2 cells could finally lead to immune escape and T cell dysfunction in high-risk patients. A previous study suggested that CXCL13 can serve to provide feedback related to the immunosuppressive microenvironment and T cell dysfunction 40. Our finding of a positive correlation between the risk score and CXCL13 indicated that CXCL13 may also engage in T cell exhaustion in the group of high-risk patients. In addition, the differently expressed LRGs were significantly enriched regarding regulation of the humoral immune response, T cell activation, macrophage differentiation, and chemokine-mediated signaling pathway. In light of these results, we investigated the expression of D2-40^+^ LVD and CD8^+^ TILs and CD163^+^ TAMs by IHC staining and found a positive correlation among D2-40^+^ LVD and CD8^+^ TILs, or CD163^+^ TAMs in the ccRCC sections of our clinical center. Moreover, increased D2-40^+^ LVD was positively associated with CD8^+^PD1^+^ TILs or CD8^+^LAG3^+^ TILs, indicating the increased CD8^+^ T cells in ccRCC patients with lymphangiogenesis may exhibited an exhausted phenotype due to the upregulation of these immunosuppressive molecules. These findings indicate that the LRG signature may impact the prognosis of ccRCC patients by interference with the TME.

Gene mutations are regarded to be a significant event that can lead to carcinogenesis, and genetic mutations represent the guiding basis for prognosis and treatment 45. In our study, higher TMB scores were observed in patients with a high-risk score. Patients in the high-risk group had a significantly elevated rate of BAP1 mutations. It has previously been reported that a mutation in BAP1 is a biomarker for both high TMB and poor prognosis in ccRCC patients 46, 47. These findings indicated that the LRG signature had a potential interaction with somatic mutations and the BAP1 mutation may be an upstream molecular event that mediates lymphoangiogenesis in ccRCC patients.

Metastatic ccRCC has a poor prognosis due to an intrinsic resistance to chemotherapy and targeted antiangiogenic therapy. Moreover, lymphangiogenesis can contribute to poor prognosis by transferring tumor cells to distant organs; however, lymphangiogenesis may also facilitate the movement of tumor antigens into the lymphatic drainage and enhance the therapeutic effect of drugs 48. In esophageal cancer, lymphangiogenesis is involved in resistance to neoadjuvant chemotherapy 48, but an opposite result was reported in breast cancer 49. To assess the effectiveness of common chemotherapeutic and anti-angiogenic agents against LRG patterns, cisplatin, gemcitabine, sorafenib, pazopanib, sunitinib, and temsirolimus were investigated in this study. The results showed that a higher risk score associated with higher IC50 of cisplatin, gemcitabine, sorafenib and pazopanib, whereas a higher risk score corresponded with a lower IC50 of sunitinib and temsirolimus. In addition, the differently expressed LRGs were significantly enriched in the cellular response to the drug, regulation of the response to the drug and VEGF signaling pathway. These findings suggest that the LRG signature affects drug sensitivity, and provided an informed decision to select clinical chemotherapy and anti-angiogenic therapy in ccRCC patients. However, this study has some limitations. This is a retrospective study. Well-designed prospective research in the future is necessary and more samples are required to verify the results. We also hope to establish an easy-to-use scoring model of LRGs expression quantity in tumor samples to evaluate risk value of the prognosis. To promote the clinical transformation, it is essential to validate our results in a prospective clinical study using single cell/nucleus ccRCC datasets and spatial transcriptomic data in the future.

Overall, our results suggested that the LRG signature might has the potential for prognostic evaluation and clinical guidance of ccRCC patients. This is the first full-scale study concerning lymphangiogenesis to develop an LRG profiling prognostic model in ccRCC patients, and investigate the correlation between the LRG signature and TME, immune status, somatic mutation, and drug sensitivity. However, the specific molecular mechanisms require further exploration and additional experimental design is needed to facilitate its clinical application in the future.

## Conclusion

In conclusion, our study constructed a novel prognostic LRG signature based on four LRGs (IL4, CSF2, PROX1, and TEK). The TME, immune status, somatic mutations, and drug sensitivity were evaluated. Our findings provide insight into obtaining a prognostic assessment and individualized drug treatment for ccRCC patients. Well-designed prospective studies are needed in the future to verify our findings.

## Supplementary Material

Supplementary figures.

Supplementary table s1.

Supplementary table s2.

Supplementary table s3.
