# Supplementary material for: A Novel Lymphangiogenesis-Related Gene Signature can Predict Prognosis and Immunosuppressive Microenvironment in Patients with Clear Cell Renal Cell Carcinoma
Source: Int J Med Sci. 2023 Apr 24;20(6):754–70. doi: 10.7150/ijms.81078 (PMC10198139; doi:10.7150/ijms.81078)
Supplement: Supplementary file 1 — Supplementary figures. [file ijmsv20p0754s1.pdf]

Genes	Hazard ratio (95% CI)	P value
APLN	0.855 (0.762-0.959)	0.007
BDNF	0.799 (0.672-0.950)	0.011
CCL5	1.126 (1.009-1.258)	0.035
CD44	1.335 (1.150-1.549)	<0.001
CDH1	0.750 (0.670-0.840)	<0.001
CDKN2A	1.343 (1.126-1.602)	0.001
CEACAM1	0.698 (0.609-0.801)	<0.001
CLEC14A	0.725 (0.652-0.808)	<0.001
COL1A1	1.150 (1.055-1.253)	0.001
CSF2	2.577 (1.946-3.413)	<0.001
CXCL12	0.875 (0.784-0.977)	0.018
CXCL5	1.169 (1.096-1.246)	<0.001
ERBB2	0.652 (0.553-0.769)	<0.001
FLT1	0.778 (0.709-0.854)	<0.001
FLT4	0.768 (0.676-0.871)	<0.001
FOXP3	1.330 (1.144-1.547)	<0.001
GATA2	0.713 (0.607-0.838)	<0.001
IGFBP1	1.120 (1.068-1.175)	<0.001
IL4	8.043 (4.536-14.260)	<0.001
ITGA4	0.842 (0.740-0.960)	0.01
KDR	0.748 (0.684-0.817)	<0.001
MCAM	0.864 (0.756-0.987)	0.031
MMP9	1.155 (1.070-1.246)	<0.001
MTOR	0.721 (0.572-0.908)	0.005
NOX4	0.771 (0.668-0.890)	<0.001
NRP1	0.797 (0.704-0.903)	<0.001
NTRK2	0.824 (0.730-0.930)	0.002
PLA2G10	0.385 (0.213-0.693)	0.001
PROX1	1.397 (1.181-1.652)	<0.001
PTGER1	1.211 (1.042-1.407)	0.013
RETN	1.211 (1.024-1.433)	0.025
SERPINE1	1.116 (1.024-1.217)	0.013
SHC1	1.452 (1.143-1.843)	0.002
SPARC	0.828 (0.714-0.961)	0.013
TEK	0.661 (0.593-0.737)	<0.001
TNFSF15	0.697 (0.554-0.876)	0.002
TWIST1	1.492 (1.234-1.804)	<0.001
TYMP	1.285 (1.083-1.525)	0.004
VASH1	0.808 (0.700-0.932)	0.003
ZIC2	1.942 (1.652-2.282)	<0.001

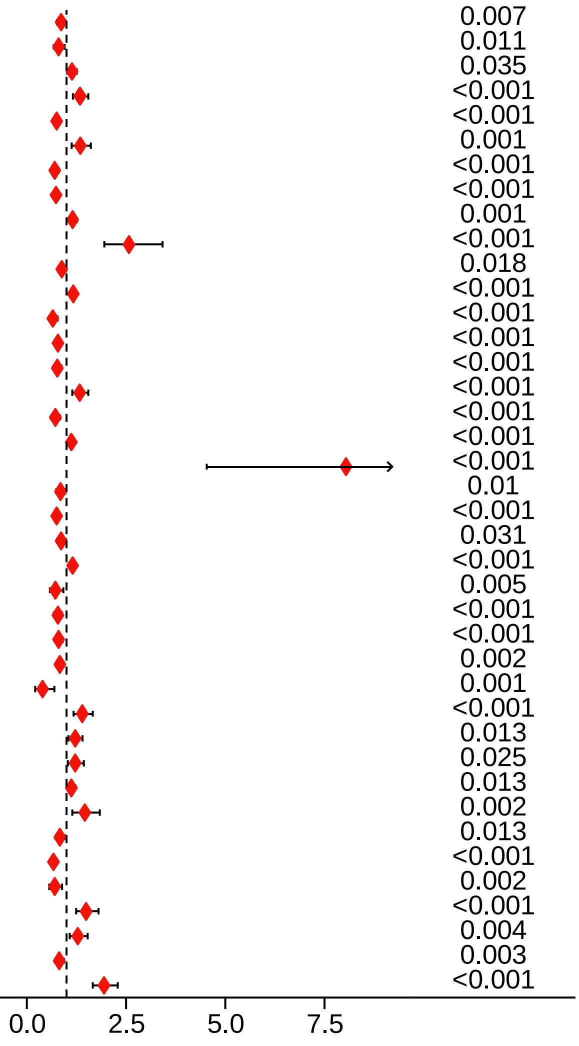

Figure S1. Forest map of the univariate Cox analysis between significant prognostic LRGs and overall survival of ccRCC patients. LRGs, lymphangiogenesis-related genes;

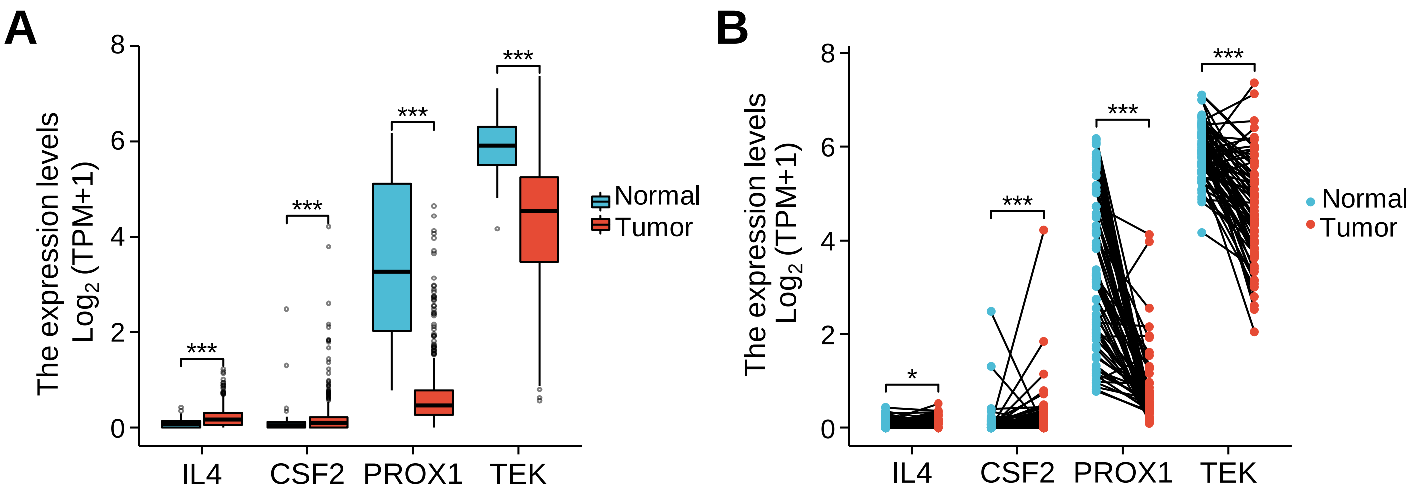

Figure S2. Differential expression of the four selected LRGs between normal and ccRCC tissue samples in the TCGA dataset. (A) The expression of the four LRGs compared between ccRCC and normal tissue. (B) Matched pairwise comparative analysis of the expression of four LRGs between ccRCC and their corresponding normal tissues. LRGs, lymphangiogenesis-related genes; *, $P < 0.05$; **, $P < 0.01$; ***, $P < 0.001$.

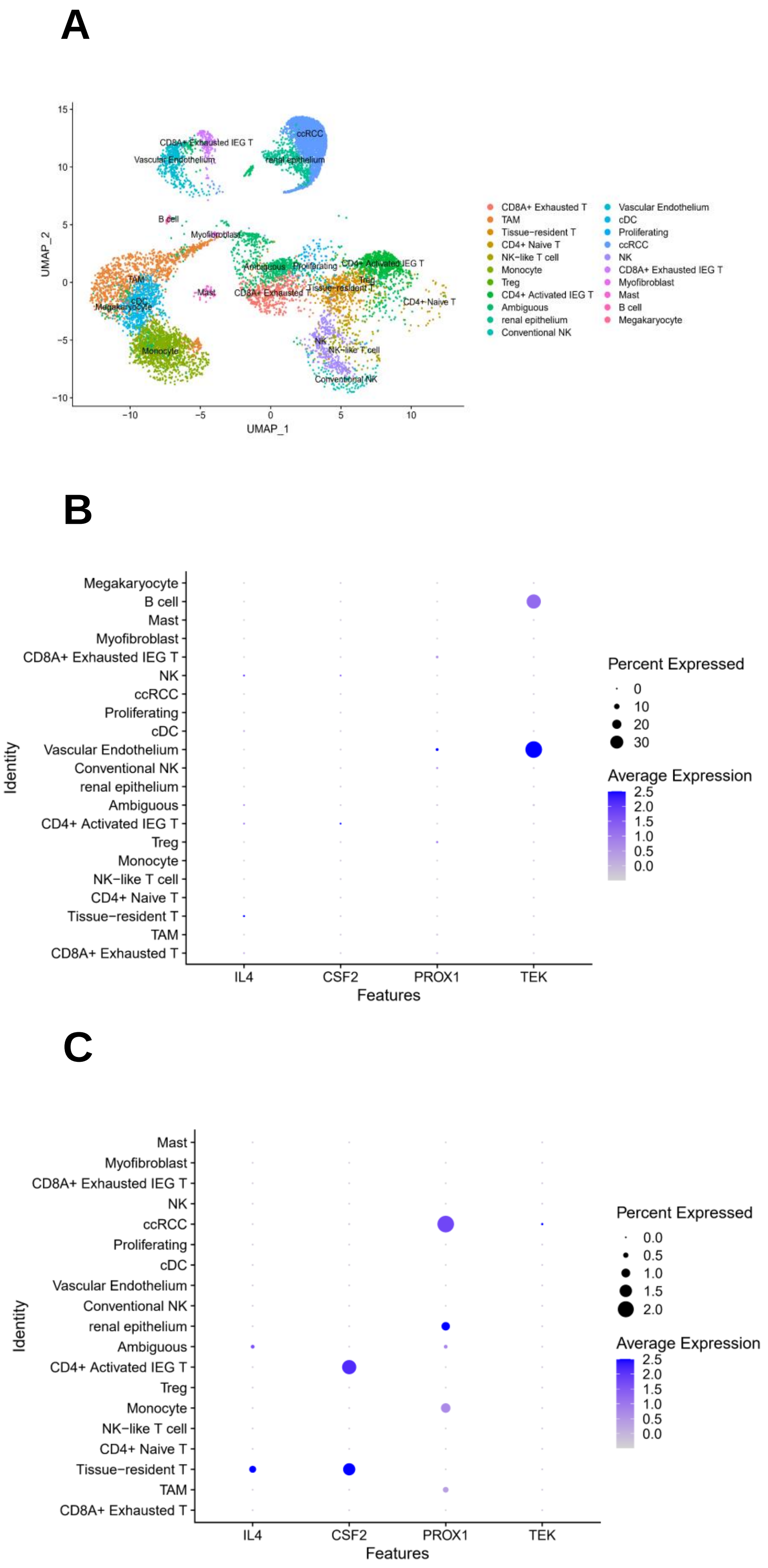

Figure S3. The selected genes expression in the ccRCC single-cell sequencing database. (A) A UMAP embedding plot of 39,390 cells grouped into 21 clusters of 2 patients named UT1 and UT2. (B, C) Dot plots showing the selected genes across all cell clusters of UT1(B) and UT2(C). The dot size indicates the fraction of expressing cells, and dots are colored according to normalized z score expression.
